# Adult allergic rhinitis sufferers have unique nasal mucosal and peripheral blood immune gene expression profiles: A case–control study

**DOI:** 10.1002/iid3.545

**Published:** 2021-10-12

**Authors:** Annabelle M. Watts, Nicholas P. West, Peter K. Smith, Allan W. Cripps, Amanda J. Cox

**Affiliations:** ^1^ Menzies Health Institute of Queensland Griffith University Southport Queensland Australia; ^2^ School of Medical Science Griffith University Southport Queensland Australia; ^3^ School of Medicine Griffith University Southport Queensland Australia; ^4^ Queensland Allergy Services Clinic Southport Queensland Australia

**Keywords:** epithelium, gene expression, mucosa, rhinitis

## Abstract

**Background:**

Allergic rhinitis (AR) is a complex disease involving both mucosal and systemic immune compartments. Greater understanding of the immune networks underpinning AR pathophysiology may assist with further refining disease‐specific biomarkers.

**Objective:**

To compare immune gene expression profiles in nasal mucosa and peripheral blood samples between adults with AR and controls without AR.

**Methods:**

This cross‐sectional study included 45 adults with moderate‐severe and persistent AR (37.6 ± 12.8 years; mean ± *SD*) and 24 adults without AR (36.6 ± 10.2). Gene expression analysis was performed using the NanoString nCounter PanCancer Immune profiling panel (*n* = 730 immune genes) in combination with the panel plus probe set (*n* = 30 allergy‐related genes) with purified RNA from peripheral blood and cell lysates prepared from combined nasal lavage and nasal brushing.

**Results:**

One hundred and thirteen genes were significantly differentially expressed in peripheral blood samples between groups (*p* < .05). In contrast, 14 genes were differentially expressed in nasal lysate samples between groups (*p* < .05). Upregulation of allergy‐related genes in nasal mucosa samples in the AR group was observed. Namely, chemokines CCL17 and CCL26 are involved in the chemotaxis of key effector cells and TPSAB1 encodes tryptase, an inflammatory mediator released from activated mast cells and basophils. Six differentially expressed genes (DEGs) were in common between the nasal mucosa and blood samples. In addition, counts of specific DEGs in nasal mucosa samples were positively correlated with eosinophil and dust mite‐specific immunoglobulin E (IgE) counts in blood.

**Conclusions and Clinical Relevance:**

Distinct gene expression profiles in blood and nasal mucosa samples were observed between AR sufferers and controls. The results of this study also provide evidence for a close interaction between the local site and systemic immunity. The genes identified in this study contribute to the current knowledge of AR pathophysiology and may serve as biomarkers to evaluate the effectiveness of treatment regimens, or as targets for drug discovery.

## Graphical abstract

A series of genes were differentially expressed in both peripheral blood and nasal mucosal samples from adults with moderate‐severe persistent allergic rhinitis compared with adults without a history of allergic rhinitis.

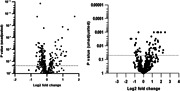

## 1 INTRODUCTION

Allergic rhinitis (AR) is classified as a chronic upper respiratory disease estimated to affect between 10% and 30% of the global population^1^, ^2^, ^3^, ^4^ and is associated with significant medical and economic burden.^5^, ^6^, ^7^ AR symptoms occur primarily in the upper respiratory tract; however, the immunopathology of the disease is highly complex and involves interactions between the local site (nasal mucosa) and the systemic immune system (lymphoid tissues and peripheral blood). Indeed, changes to the activation status of peripheral blood leukocytes were observed following nasal allergen challenge in seasonal AR sufferers.^8^ Specifically, fluctuations in the frequency of peripheral blood CD107a, CD63, and CD203c basophil activation makers, CD80( ^+^ ) and CD86(^ +^ ) plasmacytoid dendritic cells and CD4( ^+^ ) CD25lo memory T cells have been observed following nasal allergen challenge.^8^ These findings support the concept that allergen exposure at the local respiratory site can induce a systemic immunological response, particularly observed in cell types that are consistent with the AR phenotype.

Multiplex gene expression analyses, including microarray experiments, are an effective means of gaining a global representation of the cellular mechanisms behind complex diseases. Additionally, gene expression experiments are well suited to identifying genes involved in the pathophysiology of specific diseases and in identifying potential drug targets. Traditionally, microarray experiments using nasal mucosal samples have typically relied on nasal biopsy specimens^9^, ^10^ or have pooled nasal lavage samples from multiple individuals to obtain sufficient sample material for analysis.^11^ We have successfully conducted gene expression experiments with cell lysate samples collected via nasal brushing and nasal washing using the NanoString nCounter system.^12^ This method provided a cost‐effective and noninvasive means of sampling that yielded sufficient molecular material for expression analysis of a panel of 760 immune genes.

We have applied this gene expression technology to identify AR‐specific genes and characterize immune‐gene expression profiles of nasal mucosal and peripheral blood samples of participants with AR compared with a cohort of healthy non‐AR controls. The combined approach of analyzing both the local (nasal mucosa) and systemic immune system (peripheral blood) provides a powerful tool to further understand immunological networks underpinning AR pathophysiology.

## 2 METHODS

### 2.1 Study design

This study was designed as a cross‐sectional study to characterize differences in the immune gene expression profiles in peripheral blood and nasal mucosa samples between adults with existing AR (*n* = 45) and adults with no history of AR (*n* = 24 controls). Participants attended a screening appointment at the Queensland Allergy Services Clinic in Southport for evaluation of allergen sensitization and clinical characteristics. Following screening, eligible participants were instructed to cease use of all intranasal and immune modulating medications for 14 days before their follow‐up appointment. Participants then attended an appointment at the Clinical Trial Unit at Griffith University for provision of blood and nasal mucosal samples and completion of symptom surveys. This study was approved by the Griffith University Human Research Ethics Committee (approval #s: 2015/564; 2016/279) and completed in accordance with the Declaration of Helsinki. All participants provided written and informed consent before participation.

### 2.2 Participant selection

Participants were both male and female aged between 18 and 65 years of age with a more than 2‐year history of AR symptoms. Participants had persistent AR and moderate‐to‐severe symptoms as defined by the Allergic Rhinitis and Its Impact on Asthma guidelines (ARIA) with symptoms occurring for more than 4 days per week and more than 4 weeks in a row, and one or more of the following conditions present (1) sleep disturbance, (2) impairment of daily activities, (3) impairment of school or work, or (4) troublesome symptoms. Participants had a positive allergic response to dust mites determined via a skin prick test and/or serum‐specific immunoglobulin E (IgE) radioallergosorbent test (RAST) (QML Pathology) to *Dermatophagoides pternyssinus* or *Dermatophagoides farinae*. Participants were also tested against a panel of pollen allergens and an IgE RAST for grass pollen mix (Bermuda, Timothy, Meadow, Johnson, Rye, and Paspalum) for characterization of the cohort.

AR individuals were excluded from participating if they suffered from non‐AR (vasomotor rhinitis), consumed probiotics in the previous 8–12 weeks, were treated with oral corticosteroids within the previous 6 months or antibiotics within the previous 30 days, used anti‐inflammatory or immune‐modulating medications, had existing respiratory disease including asthma, nasal polyposis, or chronic obstructive pulmonary disorder, had existing immune dysfunction (other than allergies), had recent nasal surgery or nasal trauma that could affect nasal mucosal sampling, were ill or had infectious disease at the time of enrollment, reported hepatic impairment, or excessive alcohol consumption as per the NHMRC alcohol guidelines Australia^13^ and Bouchery et al.,^14^ or were pregnant at the time of enrollment.

Individuals were recruited to the study as control group (CG) if they reported no history of AR, tested negative to dust mites, grass and tree pollen, and were free from any chronic disease. Participants were excluded from the CG if they consumed probiotics in the previous 8–12 weeks, had taken antibiotics within the previous 30 days, used anti‐inflammatory or immune‐modulating medications, had existing respiratory disease, immune dysfunction or gastrointestinal disease or disorder, were ill or had an infectious disease at the time of enrollment or were pregnant at the time of enrollment.

### 2.3 Symptom analysis

The severity of AR symptoms was evaluated using a collection of self‐reported symptom surveys completed before sample collection. The AR‐specific symptom severity surveys included the mini Rhinoconjunctivitis Quality of Life Questionnaire (mRQLQ), Total nasal symptom score (TNSS) survey, Total Ocular Symptom Score (TOSS) survey, The Other Allergic Rhinitis Symptom Score (OARSS), and overall symptom severity measured using a 100 mm Visual Analogue Scale. The mRQLQ consists of 14 questions separated into five domains: activities, practical problems, nose symptoms, eye symptoms, and other symptoms. All items on the questionnaire were rated on a 7‐point likert scale (0‐6) with each item averaged to give a maximum overall score of six. The TNSS consisted of nasal congestion, runny nose, itchy nose, and sneezing. Symptoms were scored on a four‐point scale: 0, no symptoms; 1, mild symptoms; 2, moderate symptoms; and 3, severe symptoms; such that the maximum daily TNSS score was 12. The TOSS consisted of itchy eyes, watery eyes, eye redness, scored on the same four‐point scale as the TNSS such that the maximum daily TOSS was 9. The other symptom score consisted of postnasal drip, unrefreshed sleep, itchy throat/palate or ears and sinus pain, scored on the same four‐point scale, such that the daily maximum score was 12.

### 2.4 Sample collection and laboratory analysis

Venous blood samples were collected for full blood count including white cell differential and specific IgE for dust mites and grass pollen mix. In addition, erythrocyte sedimentation rate (ESR) over 1 h was measured using fresh blood samples collected in sodium citrate tubes and using commercially available Vacuette ESR pipettes (Greiner Bio‐One) as per the Westergren method.^15^

Nasal washing, brushing, and whole blood samples were collected as described previously.^12^ Briefly, nasal wash samples were collected by instilling 100 ml of phosphate‐buffered saline (PBS) in each nostril. Expelled fluid was collected in a sterile container and supplemented with 20 ml of RPMI media. Nasal brushings were collected with a brush placed between the nasal septum and inferior turbinate of each nostril. Harvested nasal mucosal cells were shaken from nasal brushes into 3.5 ml RPMI media to release cells. Nasal wash and nasal brushing samples were combined, and cellular material concentrated via centrifugation with subsequent lysis using a commercially available RLT lysis buffer (Qiagen). For whole blood samples, RNA was extracted from PAXgene tubes using a Maxwell® RSC automated RNA extraction instrument using commercially available Maxwell® RSC simplyRNA Tissue Kit (Promega Corporation). The quality and quantity of extracted RNA was assessed with the NanoDrop 1000 UV‐Vis spectrophotometer (ThermoScientific) and by capillary electrophoresis (LapChip GXII Touch HT, Perkin Elmer).

### 2.5 Gene expression analysis

Immune gene expression analysis of nasal cell lysate and extracted RNA from blood was performed using a commercially available NanoString nCounter PanCancer Immune Profiling panel (NanoString Technologies) as described previously.^12^ This panel contained 40 reference (housekeeping) genes and 730 immune genes and was used in combination with the nCounter panel plus probe set which contained an additional 30 immune genes (760 immune genes in total).^12^ Gene expression data underwent imaging quality control and normalization checks before analysis and interpretation of data. Raw NanoString gene expression data were first normalized against a set of eight negative controls to account for background noise and platform associated variation by subtracting the mean + 2 standard deviations of the negative control counts from each sample. The data were then normalized against the geometric mean of six positive control samples. Samples that did not meet the quality control parameters where removed from further analysis. Genes were removed from further analysis if they had under 20 raw counts in greater than 50% of samples from either group. The GeNorm Algorithm was used to select the most stable housekeeping genes to use for reference normalization. In total, 34 housekeeping genes were used for reference normalization of the blood samples and 29 housekeeping genes were used to perform reference normalization of the nasal lysate samples.

### 2.6 Pathway analysis

Functional gene annotation and pathway analysis was conducted using the Database for Annotation, Visualization and Integrated Discovery (DAVID) using differentially expressed genes (DEGs) (*p* < .05 *p*adjust) with significant pathway enrichment accepted at *p* < .05 Bonferroni *p*adjust.

### 2.7 Protein–protein interaction (PPI) network

The DEGs were analyzed with Search Tool for the Retrieval of Interacting Genes (STRING) (http://string-db.org/). STRING is an online database for predicting functional interactions between proteins. The minimum required interaction score was defined at 0.40.

### 2.8 Statistical analysis

Differences in demographic and clinical measures between groups was assessed with an independent *t* test for continuous variables and a *χ* ^2^ test for categorical variables. Differential gene expression analysis was performed on normalized data using the nSolver Advanced analysis software version 4.0 (NanoString technologies) with the Bonferroni Hochberg *p* value correction (*p*adjust) with significance accepted at *p* < .05. Correlations between clinical features and DEG counts were performed using the Pearson correlation coefficient with significance accepted at *p* < .05.

## 3 RESULTS

### 3.1 Study participants

The demographic and clinical characteristics of the cohort are included in Table 1. The groups were matched in key physical attributes. However consistent with diagnosis of atopic conditions, the AR group had significantly higher white blood cell, lymphocyte, eosinophil, and basophil counts compared with the CG. The AR group had moderate symptoms based on the symptom severity questionnaires, with the majority sensitized to both plant pollen and dust mites (Table 2). Several participants also reported allergies other than AR; a total of 49% of the cohort reported a history of skin allergies (eczema, hand dermatitis, urticaria, and itchy rash), 27% also reported a history of food allergy, and 11% also reported a history of drug allergy (including Codeine [opioid], antibiotics, and metoclopramide [dopamine D_2_ receptor antagonist/5‐HT_3_ receptor antagonist/5‐HT_4_ receptor agonist]). Nasal lysate samples from 37 AR participants and 21 CG participants met the quality control guidelines and were included in gene expression analysis. The demographic and clinical characteristics of the participants whose nasal lysate samples were included in the gene expression analysis were similar to that of the entire study cohort and are included in Tables S1 and S2.

**Table 1:** Clinical and demographic features of the study cohort

	AR mean ± *SD*	CG mean ± *SD*	*p* value
n	45	24	–
Age (years)	37.58 ± 12.82	36.57 ± 10.22	.74
Sex (M/F)	16/29 (64% F)	11/13 (54% F)	.405
Height (cm)	171.30 ± 9.33	175.11 ± 9.52	.113
Weight (kg)	74.40 ± 15.11	75.51 ± 19.40	.808
BMI (kg/m^2^)	25.18 ± 3.73	24.34 ± 4.63	.411
Ethnicity (% Caucasian)	78%	96%	.051
Immune measures			
White cell count (x10^9^/L)	6.60 ± 1.82	5.58 ± 1.27	.017
Lymphocytes (x10^9^/L)	2.18 ± 0.73	1.83 ± 0.59	.045
Eosinophils (x10^9^/L)	0.41 ± 0.30	0.11 ± 0.08	<.00001
Neutrophils (x10^9^/L)	3.45 ± 1.15	3.15 ± 0.97	.273
Basophils (x10^9^/L)	0.06 ± 0.04	0.04 ± 0.03	.025
ESR (mm/hr)	9.11 ± 9.11	7.38 ± 7.64	.429

Abbreviations: %, percentage; AR, allergic rhinitis; BMI, body mass index; cm, centimeter; CG, control group; ESR, erythrocyte sedimentation rate; F, female; hr, hour; kg, kilogram; L, litre;m, meter; M, Male; n, number; mm, millimeter.

**Table 2:** Disease characteristics of the primary AR cohort (blood samples)

Disease characteristic	AR (mean ± *SD*)
Allergen sensitivity	
Coallergy to dust mites and pollen (%)	60%
Dust mite only (%)	40%
IgE *Dermatophagoides pteronyssinus* (kU/L)	24.05 ± 31.94
IgE *D. farinae* (kU/L)	19.90 ± 29.21
IgE grass pollen mix (kU/L)	7.30 ± 20.33
IgG4 *D. pteronyssinus* (kU/L)	0.46 ± 0.46
IgG4 *D. farinae* (kU/L)	0.37 ± 0.35
IgG4 grass pollen mix (kU/L)	0.86 ± 0.72
Symptom severity	
Total nasal symptom score (0–12 U)	5.4 ± 3.26
Total ocular symptom score (0–9 U)	2.71 ± 2.40
Mini rhinoconjunctivitis quality of life score (0–6 U)	2.8 ± 1.06
Other allergic rhinitis symptom score (0–12 U)	4.04 ± 3.27
Visual analogue scale (0–100 mm)	52.47 ± 27.94

Abbreviations: %, percentage; AR, allergic rhinitis; IgE, immunoglobulin E; kU, kilounit; L, Litre; mm, millimeter; U, unit.

### 3.2 DEGs between AR and non‐AR controls

#### 3.2.1 Blood

**Figure 1:**
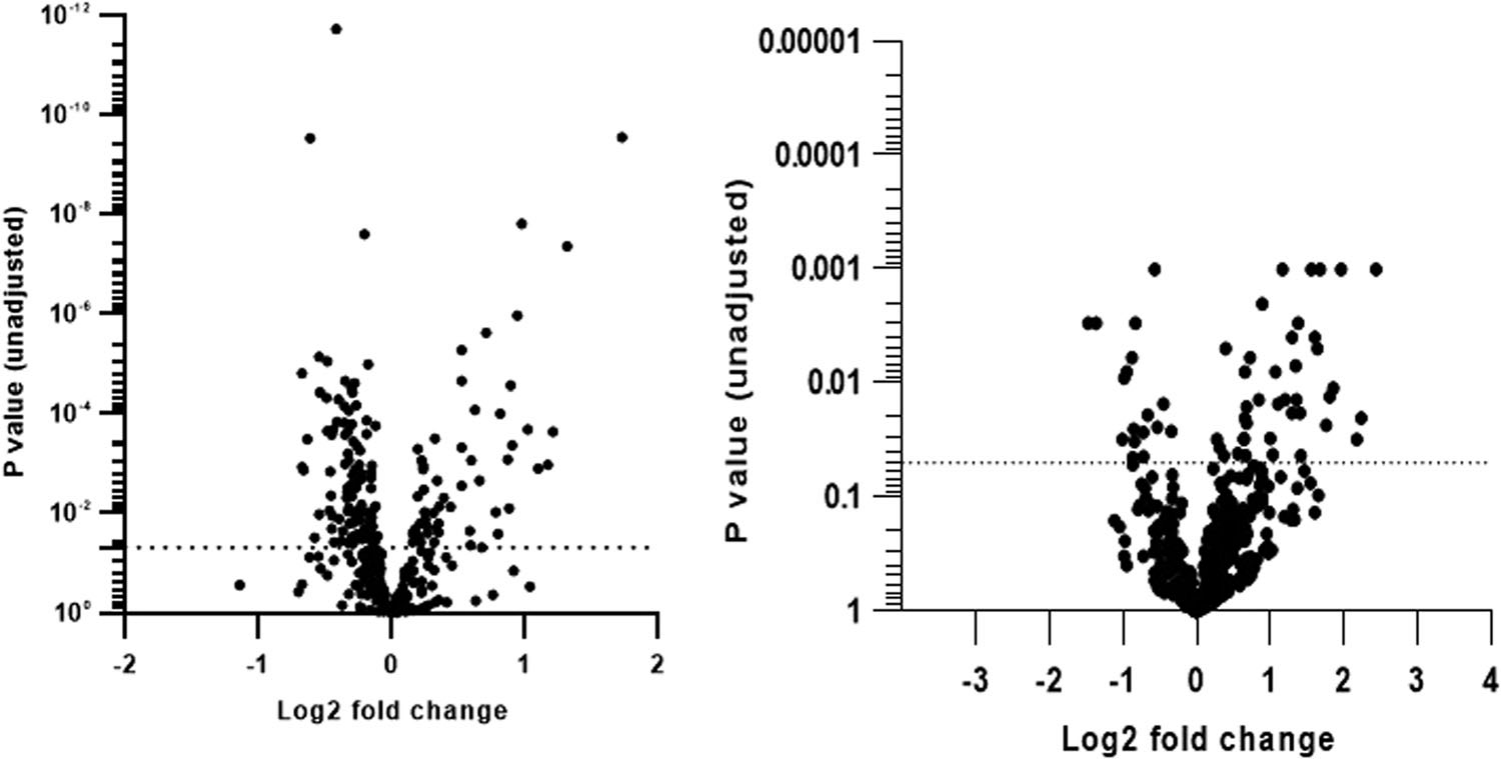
Volcano plot summarizing the differential gene expression. The blood samples are on the left panel and the nasal lysate samples are on the right panel. Fold changes greater than zero indicate increased gene expression in AR samples compared with the CG. Dotted horizontal line indicates significance at *p* value .05

Of the 760 immune genes tested on the NanoString arrays, 466 were expressed above background noise and were included in the subsequent analyses. Gene expression changes in all genes are summarized in Figure 1 (left panel). In total, 175 genes were differentially expressed between the AR and CG cohorts based on *p* < .05 and 113 genes were differentially expressed after controlling for false discovery rate (FDR) (*p*adjust) (Table S3). The top 20 DEGs according to *p* value are shown in Table 3. Of the 113 DEGs, 35 genes were upregulated (1.73 Log2 FC to 0.195 Log2 FC) and 78 genes were downregulated compared with the CG (−0.674 log2 to −0.119 Log2 FC).

**Table 3:** Top 20 differentially expressed genes for blood and nasal lysate samples

Gene	Log2 fold change	Linear fold change	Lower confidence limit (log2)	Upper confidence limit (log2)	*p* value	*p* adjust
Blood						
MAP2K1	−0.417	0.749	−0.512	−0.322	1.84 × 10^−12^	9.86 × 10^−10^
TBK1	−0.608	0.656	−0.769	−0.447	2.90 × 10^−10^	5.18 × 10^−8^
PTGDR2	1.730	3.310	1.270	2.180	2.78 × 10^−10^	5.18 × 10^−8^
CD83	0.979	1.970	0.681	1.280	1.53 × 10^−8^	1.65 × 10^−6^
CD164	−0.203	0.869	−0.266	−0.140	2.47 × 10^−8^	2.21 × 10^−6^
CD24	1.320	2.500	0.903	1.740	4.22 × 10^−8^	3.24 × 10^−6^
IL2RA	0.946	1.930	0.601	1.290	1.06 × 10^−6^	7.12 × 10^−5^
MICA	0.711	1.640	0.441	0.980	2.31 × 10^−6^	1.38 × 10^−4^
IL12RB1	0.527	1.440	0.318	0.736	5.31 × 10^−6^	2.85 × 10^−4^
ABCB1	−0.544	0.686	−0.764	−0.325	7.21 × 10^−6^	3.52 × 10^−4^
LILRA1	−0.484	0.715	−0.681	−0.287	8.71 × 10^−6^	3.90 × 10^−4^
HMGB1	−0.177	0.884	−0.250	−0.104	1.02 × 10^−5^	4.23 × 10^−4^
NCR1	−0.674	0.627	−0.958	−0.391	1.51 × 10^−5^	5.79 × 10^−4^
IFNGR1	−0.350	0.785	−0.500	−0.200	2.20 × 10^−5^	7.40 × 10^−4^
IRF3	0.527	1.440	0.300	0.753	2.20 × 10^−5^	7.40 × 10^−4^
TNFAIP3	−0.275	0.827	−0.393	−0.156	2.42 × 10^−5^	7.65 × 10^−4^
HRH4	0.896	1.860	0.507	1.280	2.64 × 10^−5^	7.88 × 10^−4^
IKBKG	−0.303	0.811	−0.436	−0.171	2.94 × 10^−5^	8.31 × 10^−4^
APP	−0.536	0.690	−0.774	−0.298	3.68 × 10^−5^	9.08 × 10^−4^
PSEN1	−0.292	0.817	−0.422	−0.163	3.61 × 10^−5^	9.08 × 10^−4^
Nasal lysate						
CCL17	2.84	7.14	2.03	3.64	4.41 × 10^−9^	2.51 × 10^−6^
CCL26	2.72	6.61	1.82	3.63	2.39 × 10^−7^	5.13 × 10^−5^
TPSAB1	3.08	8.45	2.05	4.11	2.71 × 10^−7^	5.13 × 10^−5^
PTGS1	2.41	5.30	1.49	3.33	4.01 × 10^−6^	4.57 × 10^−4^
IL1RL1	3.41	10.70	1.97	4.86	2.14 × 10^−5^	2.02 × 10^−3^
CD1A	2.46	5.52	1.40	3.53	3.05 × 10^−5^	2.48 × 10^−3^
CCND3	1.13	2.19	0.57	1.69	2.36 × 10^−4^	1.68 × 10^−2^
PPBP	−1.65	0.32	−2.51	−0.78	4.60 × 10^−4^	2.91 × 10^−2^
IL18R1	1.96	3.89	0.88	3.04	7.53 × 10^−4^	3.89 × 10^−2^
CD1C	1.56	2.96	0.68	2.44	9.70 × 10^−4^	4.04 × 10^−2^
CTSH	−0.57	0.68	−0.89	−0.25	1.01 × 10^−3^	4.04 × 10^−2^
FLT3LG	1.16	2.23	0.50	1.81	1.06 × 10^−3^	4.04 × 10^−2^
RUNX3	1.68	3.21	0.74	2.63	9.63 × 10^−4^	4.04 × 10^−2^
PTGDR2	2.44	5.43	1.04	3.84	1.17 × 10^−3^	4.15 × 10^−2^
IL13	1.61	3.06	0.63	2.59	2.11 × 10^−3^	6.66 × 10^−2^
JUN	−0.83	0.56	−1.34	−0.31	2.63 × 10^−3^	7.14 × 10^−2^
TXNIP	0.89	1.85	0.34	1.44	2.45 × 10^−3^	7.14 × 10^−2^
KLRB1	−1.47	0.36	−2.39	−0.55	2.78 × 10^−3^	7.20 × 10^−2^
CXCR3	1.38	2.60	0.51	2.25	3.06 × 10^−3^	7.58 × 10^−2^
CARD9	1.29	2.45	0.46	2.12	3.50 × 10^−3^	8.31 × 10^−2^

#### 3.2.2 Nasal lysate

A total of 474 immune genes were expressed above background noise and were included in the subsequent analyses. Gene expression changes in all genes are summarized in Figure 1 (right panel). In total, 63 genes were differentially expressed between the AR and CG cohorts based on *p* < .05 and 14 genes were differentially expressed after controlling for FDR (Table S4). The top 20 DEGs according to *p* value are shown in Table 3. Of the 14 DEGs, 12 genes were upregulated (1.13 Log2 FC to 3.41 Log2 FC) and 2 genes were downregulated compared with the CG (−1.65 log2 to −0.568 Log2 FC).

### 3.3 Enrichment of the DEGs into pathways

#### 3.3.1 Blood

The DEGs were significantly enriched into 66 Kyoto Encyclopedia of Genes and Genomes (KEGG) pathways. The top four KEGG pathways were toll‐like receptor signaling pathway, cytokine–cytokine receptor interaction, osteoclast differentiation, and chagas disease (American trypanosomiasis) (Table 4). The DEGs were also enriched into 189 DAVID genetic association database (GAD) disease pathways. The top four GAD disease pathways include type 2 diabetes/edema/rosiglitazone, respiratory syncytial virus bronchiolitis, asthma/bronchiolitis, viral/respiratory syncytial virus infections, and bronchiolitis, viral/respiratory syncytial virus infections (Table 5).

#### 3.3.2 Nasal lysate

The DEGs were significantly enriched into three KEGG pathways. These KEGG pathways include cytokine–cytokine receptor interaction, hematopoietic cell lineage, and chemokine signaling pathway (Table 4). The DEGs were also enriched into 17 DAVID GAD pathways. The top four GAD disease pathways include asthma, asthma/bronchiolitis, viral/respiratory syncytial virus infections, respiratory syncytial virus bronchiolitis and bronchiolitis, viral/respiratory syncytial virus infections (Table 5).

**Table 4:** Enriched KEGG pathways blood and nasal lysate samples

KEGG pathway	Count	Percentage	*p* value	Adjust. *p* value (Benjamini)
Blood				
Cytokine–cytokine receptor interaction	23	20.4	4.3 × 10^−12^	3.5 × 10^−10^
Osteoclast differentiation	18	15.9	4.9 × 10^−12^	2.7 × 10^−10^
Toll‐like receptor signaling pathway	17	15.0	2.0 × 10^−12^	3.3 × 10^−10^
Chagas disease (American trypanosomiasis)	16	14.2	2.1 × 10^−11^	8.5 × 10^−10^
Nasal lysate				
Cytokine–cytokine receptor interaction	4	28.6	3.1 × 10^−3^	6.6 × 10^−2^
Hematopoietic cell lineage	3	21.4	5.4 × 10^−3^	5.8 × 10^−2^
Chemokine signaling pathway	3	21.4	2.3 × 10^−2^	1.6 × 10^−1^

Abbreviation: KEGG, Kyoto Encyclopedia of Genes and Genomes.

**Table 5:** GAD disease pathways blood and nasal lysate samples

GAD disease pathways	Count	Percentage	*p* value	Adjust *p* value (Benjamini)
Blood				
Type 2 Diabetes| edema | rosiglitazone	56	49.6	2.1 × 10^−16^	6.3 × 10^−14^
Respiratory syncytial virus bronchiolitis	28	24.8	1.8 × 10^−25^	2.1 × 10^−22^
Asthma|Bronchiolitis, Viral|Respiratory Syncytial Virus Infections	27	23.9	4.3 × 10^−24^	2.4 × 10^−21^
Bronchiolitis, Viral|Respiratory Syncytial Virus Infections	27	23.9	1.0 × 10^−23^	2.4 × 10^−21^
Nasal lysate				
Asthma	5	35.7	8.6 × 10^−4^	5.3 × 10^−2^
Asthma/Bronchiolitis, Viral/Respiratory Syncytial Virus Infections	4	28.6	9.8 × 10^−4^	4.0 × 10^−2^
Respiratory syncytial virus bronchiolitis	4	28.6	9.8 × 10^−4^	4.0 × 10^−2^
Bronchiolitis, Viral/Respiratory Syncytial Virus infection	4	28.6	1.1 × 10^−3^	3.3 × 10^−2^

Abbreviation: GAD, genetic association database.

### 3.4 Investigation of the DEGs with protein–protein interaction networks

#### 3.4.1 Blood

**Figure 2:**
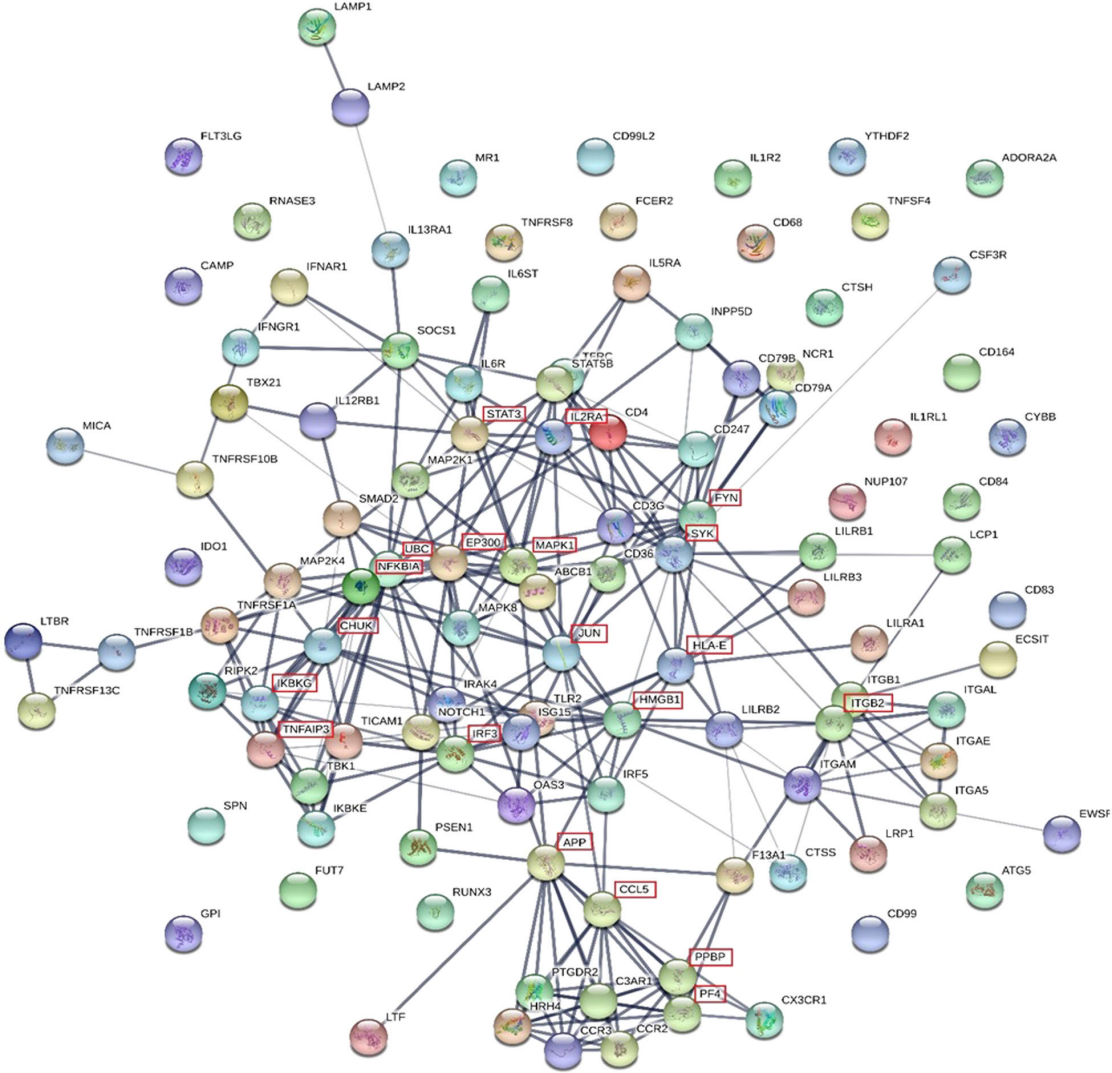
The protein–protein interaction network of differentially expressed genes in blood samples. The line thickness of the network edges indicates the strength of confidence in the interaction (the thicker the line, the greater the confidence in the interaction). Filled nodes; some 3D structure is known or predicted. Empty nodes; proteins of unknown 3D structure. The top 20 nodes are shown by a red box outlining the gene name

The DEG list was submitted to the STRING database to provide a better understanding of the biological relationship between the DEGs. A total of 112 genes were included in the PPI network with an average node degree of 4.98. As shown in Figure 2, the PPI is very complex and shows a high level of interaction between the DEGs. The top 20 nodes are listed in Table 6.

**Table 6:** Top 20 genes in the protein–protein Interaction network by degree (blood samples)

Gene	Interactions/degree
UBC	19
MAPK1	15
APP	14
EP300	14
JUN	14
CHUK	13
NFKBIA	13
STAT3	13
FYN	12
IL2RA	12
IRF3	12
ITGB2	12
CCL5	11
HLA‐E	11
HMGB1	11
IKBKG	11
PF4	10
PPBP	10
SYK	10
TNFAIP3	10

#### 3.4.2 Nasal lysate

**Figure 3:**
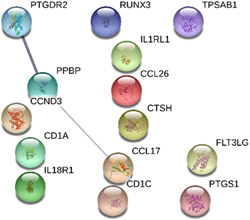
The protein–protein interaction network of differentially expressed genes (nasal lysate). The line thickness of the network edges indicates the strength of confidence in the interaction (the thicker the line, the greater the confidence in the interaction). Filled nodes; some 3D structure is known or predicted. Empty nodes; proteins of unknown 3D structure

A total of 14 genes were included in the PPI network (Figure 3) with an average node degree of 0.286. Limited interactions between the DEGs was observed. As shown in Figure 3, a single interaction pathway between PPBP and PTGDR2 and CCL17 was detected.

### 3.5 Investigation of the relationship between DEGs and clinical markers

#### 3.5.1 Blood

**Figure 4:**
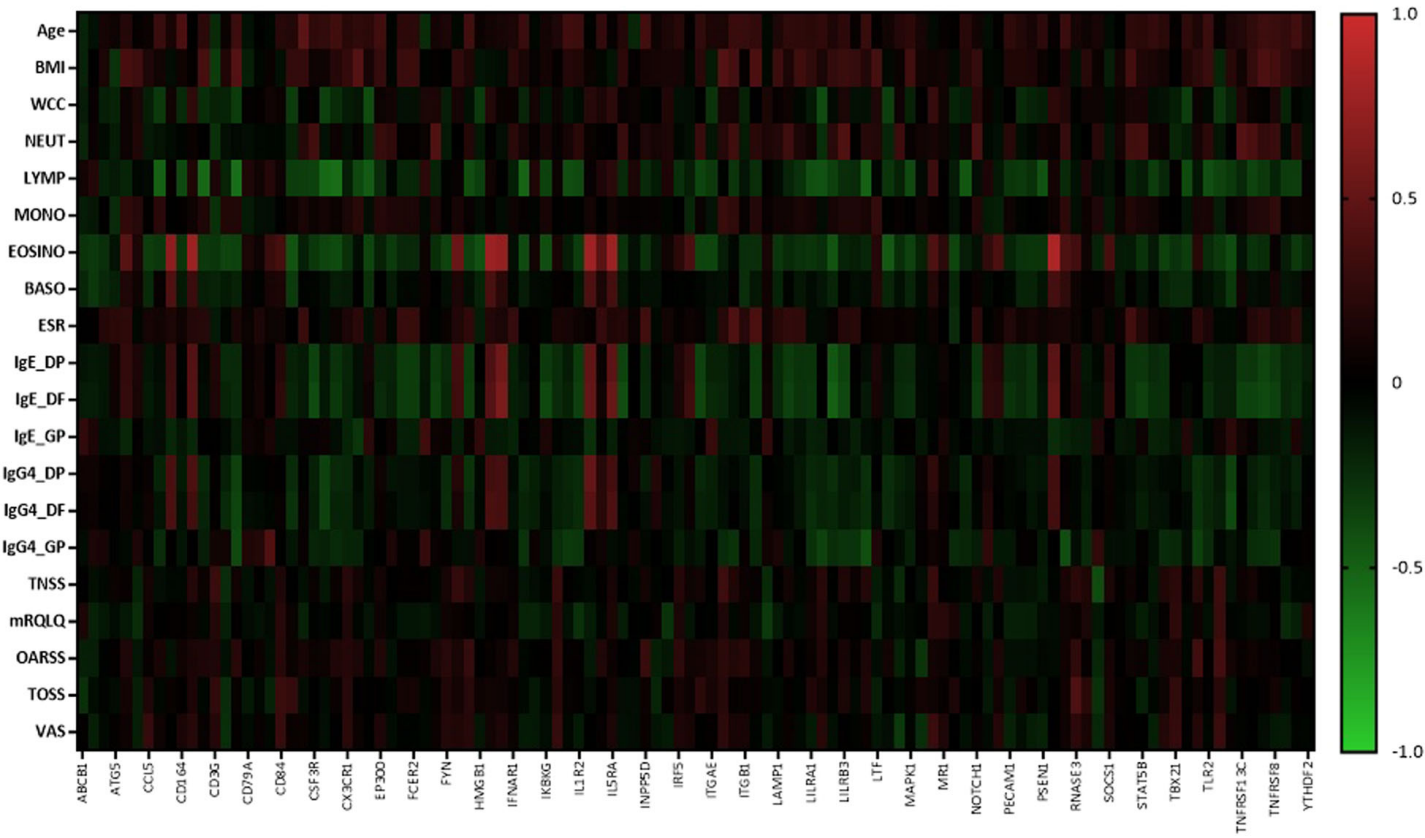
Heat map of the Pearson correlation values for clinical markers versus DEGs in blood samples. BMI, body mass index; BASO, basophil count; DEGs, differentially expressed genes; Dermatophagoides pternyssinus; DF, Dermatophagoides farinae; ESR, erythrocyte sedimentation rate; EOSINO, eosinophil count; GP, Grass pollen mix; MONO, monocyte count; MRQLQ, mini rhinoconjunctivitis quality of life questionnaire; NEUT, neutrophil count; OARSS, other allergic rhinitis symptom score; TNSS, total nasal symptom score; TOSS, total ocular symptom score; VAS, visual analogue scale; WCC, white cell count

The relationship between clinical measures and the DEGs were explored. As shown in Figure 4, eosinophils were on average moderately correlated with the DEGs (mean *R* = 0.30). Whole blood cell counts of total white cells, eosinophils, neutrophils, and lymphocytes as well as specific IgE and IgG4 were on average weakly (mean *R* = 0.1–3) associated with the DEG counts. In contrast, self‐reported symptom severity was very weakly (mean *R* = 0–1) associated with the DEGs. The top 10 individual correlations are shown in Figure S1.

#### 3.5.2 Nasal lysate

**Figure 5:**
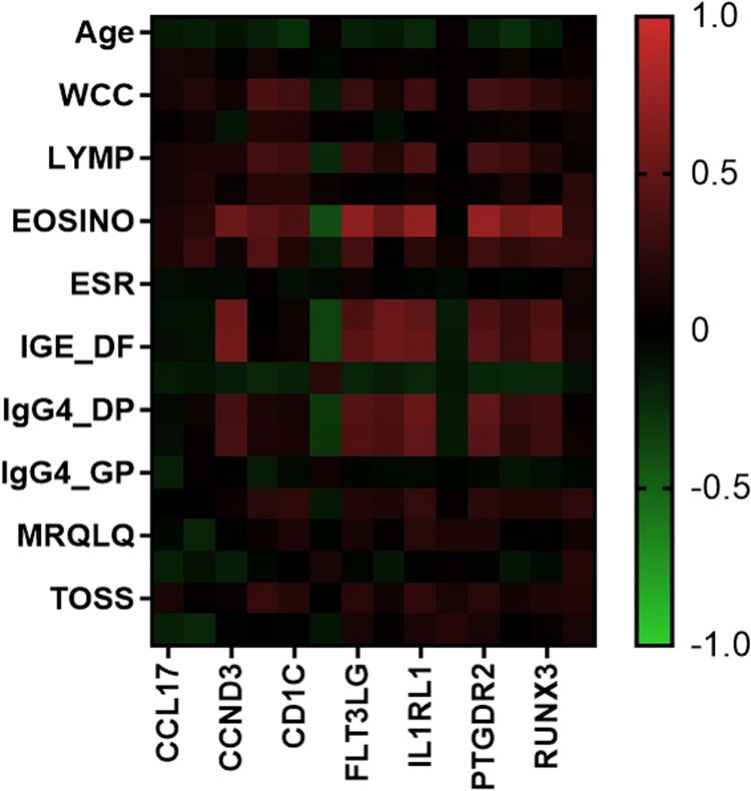
Heat map of the Pearson correlation values for clinical markers versus DEGs in nasal lysate samples. BMI, body mass index; BASO, basophil count; BASO, basophil count; DEGs, differentially expressed genes; DP *Dermatophagoides pternyssinus;* DF, *Dermatophagoides farinae;* EOSINO, eosinophil count; ESR, erythrocyte sedimentation rate; GP, Grass pollen mix; MONO, monocyte count; MRQLQ, mini rhinoconjunctivitis quality of life questionnaire; NEUT, neutrophil count; OARSS, other allergic rhinitis symptom score; TNSS, total nasal symptom score; TOSS, total ocular symptom score; VAS, visual analogue scale; WCC, white cell count

**Figure 6:**
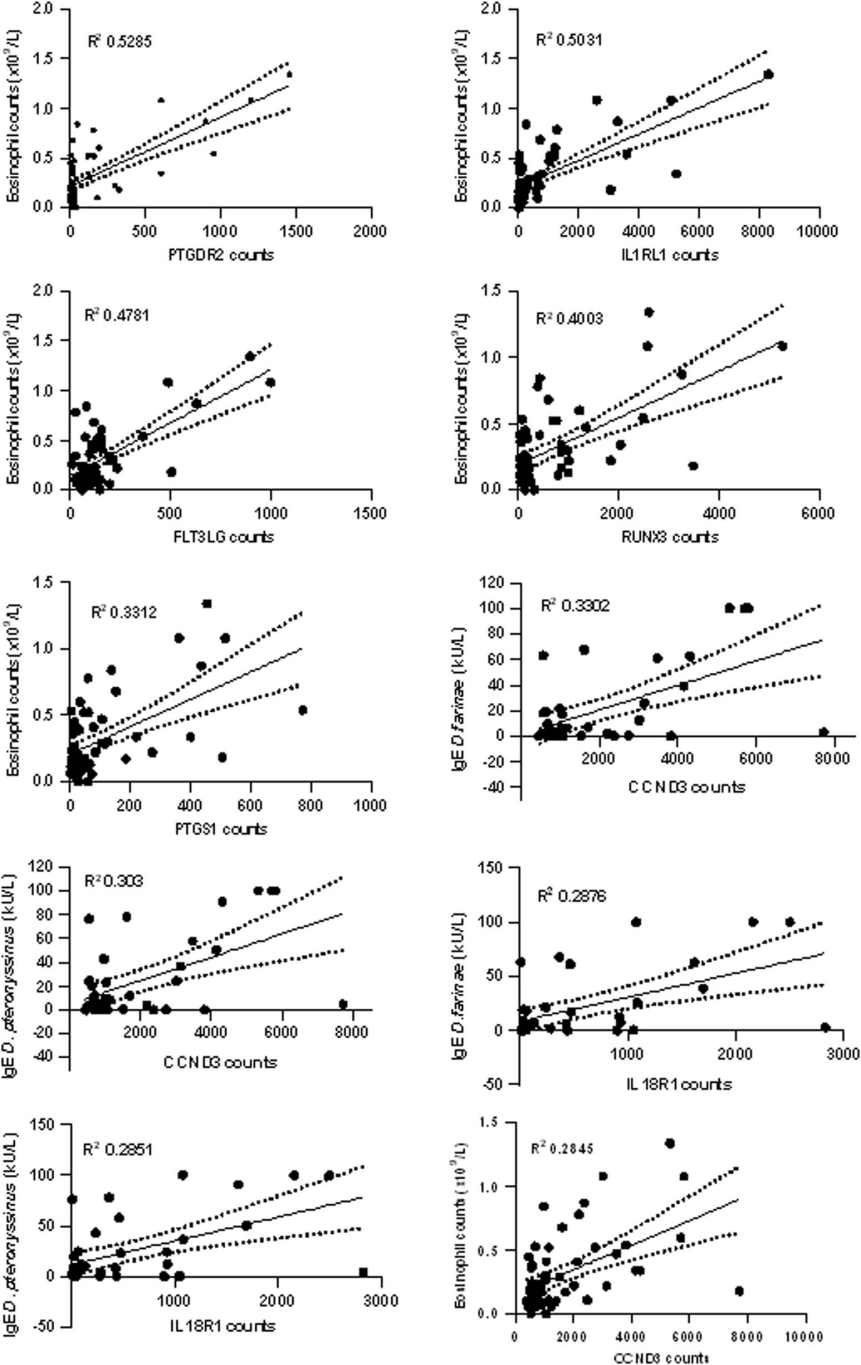
Top 10 individual correlation plots for the differentially expressed genes in nasal lysate samples versus clinical factors. Each correlation was significant (*p* < .05). Correlations for IgE *Dermatophagoides farinae* and IgE *Dermatophagoides pteronyssinus* were conducted on AR samples only (*n* = 37). The remaining correlations were performed with the inclusion of the control group samples (*n* = 58 total). AR, allergic rhinitis

Correlation analysis between the clinical factors and the nasal lysate DEGs was also performed. As shown in Figure 5, gene expression in the nasal lysate samples was on average moderately correlated with eosinophils (mean *R* = 0.45) and weakly correlated with total white cells (*R* = 0.22), lymphocytes (*R* = 0.22), and basophils (*R* = 0.22). In addition, the DEGs in nasal lysate samples were on average weakly correlated with specific IgE for dust mites (*R* = 0.28 and 0.29) and IgG4 for dust mites (*R* = 0.25 and 0.26). None or very weak (*R* = 0–0.1) correlations between BMI, neutrophils, monocytes, ESR, and self‐reported symptom severity and DEG counts was observed. The top 10 individual correlations are shown in Figure 6.

## 4 DISCUSSION

The results presented here provide further insights into the pathophysiology of AR at both the local site of the allergic response (nasal mucosa) and the systemic immune system. In this study, genes and pathways that separate AR individuals from those without AR were identified and can be explored as potential biomarkers of disease. This study provides new information about the pathophysiology of AR through the identification of genes that have not been previously associated with AR or atopy. In addition, this study supports the findings of previous studies and confirms the role of specific genes encoding prostaglandin receptors, chemokines, and eotaxin, and tryptase in the pathophysiology of AR. Evidence for interactions between the mucosal and systemic immune system during active disease is also provided in this study.

A key finding of this study was the large proportion of DEGs in blood samples from AR sufferers compared with non‐AR controls who were recruited specifically based on extensive clinical characterization of disease history, pathology confirmation of responsiveness to a single aeroallergen class (dust mites), and without the confounding effects of different intranasal medication use. A total of 113 DEGs were identified in blood samples which represents over 24% of genes that were expressed above background, thereby indicating that the gene‐expression profiles of blood samples from AR sufferers is vastly different from that of the control samples. In contrast, only 14 DEGs (~3% of genes expressed above background) were observed between the groups in the nasal mucosal samples. The relatively low number of DEGs in nasal lysate samples compared with blood samples is likely due to the large variability in mRNA expression across nasal lysate samples. The total mRNA quantity, quality, and numbers of infiltrating immune cells varied across samples and this occurrence has been previously reported using this methodology.^12^ While collection of homogeneous nasal mucosal samples across participants and repeated testing is technically challenging, sufficient molecular material for gene expression analyses was achieved using this methodology.

The top three DEGs that separate AR participants from non‐AR participants in the blood samples of patients with AR include Mitogen‐Activated Protein Kinase 1 (MAPK1), TANK binding kinase 1 (TBK1), and the Prostaglandin D2 receptor (PTGDR2), suggesting their likely involvement in the pathophysiology of AR. Both MAPK1 and TBK1 genes were downregulated in blood samples from AR participants compared with the controls. Notable functions include TBK1 which is associated with the activation and nuclear translocation of the NFκB transcription factor complex^16^ to induce the transcription of many of proinflammatory genes. Given this, the downregulation of TBK1 in AR samples was an unexpected result. In contrast, the PTGDR2 gene was significantly upregulated in both blood and nasal lysate samples and is known to play a role in the activation and chemotaxis of key inflammatory cells.^17^ Increased numbers of effector cells in the nasal mucosa would likely lead to worsened symptoms. The top three DEGs in nasal lysate samples included two CC‐chemokines (CCL17 and CCL26) and TPSAB1. These genes have been previously associated with allergic disease. CCL17 (also known as TARC) and CCL26 (also known as Eotaxin‐3) are involved in the chemotaxis of Th2 cells, and eosinophils and basophils, respectively.^18^, ^19^ The upregulation of CCL17 and CCL26 gene expression in this study is consistent with increased protein production of these chemokines reported in atopic cohorts^20^, ^21^ and in seasonal AR sufferers compared with nonallergic controls.^21^ The TPSAB1 gene encodes tryptase (alpha‐1 and beta‐1) enzymes which are released from mast cells and basophils upon activation. Increased tryptase levels have been reported in nasal lavage samples from seasonal AR sufferers following nasal allergen challenge.^22^

In this study, we identified DEGs that were unique to either nasal lysate samples or blood samples and genes that were differentially expressed in both sample types (Figure S2). A large proportion of the nasal lysate DEGs (6 of 8, 75%) including RUNX3, IL1RL1, PTGDR2, FLT3LG, CTSH, and PPBP were also significantly differentially expressed in blood samples. The common DEGs in both sample types are involved in the differentiation of multiple cell lineages and receptors/mediators involved in the chemotaxis of effector cells involved in the allergic response. The DEGs unique to nasal lysate samples are related to mediator release from mast cells, antigen presentation to T cells and chemokine release to induce chemotaxis of inflammatory cells. The DEGs unique to blood samples are primarily involved in cytokine signaling, toll‐like receptor cascades, and neutrophil degranulation. Collectively, these findings indicate that the nasal mucosal immune system and systemic immune system potentially have both individual and shared roles in the pathogenesis of AR.

The DEGs in blood and nasal lysate samples were further analyzed for enrichment into KEGG and GAD pathways. The DEGs in blood were enriched into 66 KEGG pathways. Of these enriched KEGG pathways, the top four pathways included cytokine–cytokine receptor interaction, toll‐like receptor signaling, Chagas disease, and osteoclast differentiation. These results were not entirely unexpected given that atopic diseases have been previously associated with cytokine–cytokine receptor interaction, toll‐like receptor signaling, osteoclast differentiation and pathways related to parasitic infections.^23^, ^24^, ^25^, ^26^ Interestingly, the DEGs from the nasal lysate samples were enriched into similar immune pathways as the blood samples including cytokine–cytokine receptor interaction and chemokine–chemokine signaling. The DEGs from the nasal lysate samples were also enriched into the hematopoietic cell lineage pathway which is consistent with the role of the nasal olfactory epithelium as a source of progenitor cells^27^ and the airway allergic response stimulates the production of effector cells. Interestingly, the GAD pathway analysis revealed that both the DEGs from the blood samples and nasal lysate samples were enriched in disease pathways primarily affecting the respiratory system. The top four enriched disease pathways for both the blood and nasal lysate samples included asthma, bronchiolitis, and viral respiratory disease. These data suggest that both sample types may be used as markers to identify respiratory allergic disease as well as other conditions affecting the airways.

Protein–Protein Interaction networks predict direct and indirect functional interactions between expressed proteins and provide a system‐wide understanding of cellular function under selected conditions. The DEGs in nasal lysate and blood samples were analyzed with the STRING database to identify the key genes as drivers of the allergic response. The top three genes in the protein–protein interaction network for blood samples based on degree were UBC, MAPK1 and APP, and have been previously associated with atopic disease. The metalloproteinase 33 enzyme encoded by the ADAM33 gene is a recognized allergy candidate gene and is known to cleave peptides of the amyloid precursor protein (APP).^28^ Similarly, the UBC and MAPK1 genes have been linked with asthma, which is a common comorbidity of AR.^29^ The PPI network of the nasal lysate DEGs had fewer interactions (and average node degree) than the blood DGE network. A single interaction between pro‐platelet basic protein (PPBP) and prostaglandin D2 receptor 2 (PTGDR2) and chemokine (C‐C motif) ligand (CCL17) was observed. The role of PTGDR2 and CCL17 (TARC) in the pathogenesis of allergic disease is well‐recognized.^30^, ^31^ The PPBP gene is a known chemoattractant and activator of neutrophils^32^ and its downregulation in the nasal mucosa of AR participants has not been previously reported.

The relationships between clinical measures, including white cell differential, specific IgE and IgG4, ESR, demographic features and symptom severity, and the DEG counts in blood and nasal lysate samples were explored to provide a greater understanding of the role of these genes in the pathogenesis of AR. None of the patient‐reported symptom severity measures (mRQLQ, VAS, TNSS, TOSS and OARSS) correlated with counts of the DEGs in both blood and nasal lysate samples. Response bias in self‐reported data is well‐recognized event known to confound data interpretation^33^ and may explain the lack of correlation observed between the subjective symptom severity and gene expression markers. Indeed, self‐reported symptoms and quality of life have been shown to have poor associations with objective measures of disease severity in chronic rhinosinusitis.^34^, ^35^ In contrast, moderate correlations between the blood and nasal DEGs and other clinical markers, including peripheral blood immune cell counts and serum‐specific IgE and IgG4 levels, were observed. Overall, the association between clinical markers in blood and counts of DEGs in the nasal mucosa, provide greater support for an interaction between the systemic and mucosal immune system in the pathogenesis of AR. Changes in the activation of peripheral blood leukocytes and peripheral immune gene signatures have been observed previously following local nasal allergen challenge, indicating that allergen exposure at the local mucosal site can promote changes to at the systemic immune level.^8^, ^36^ These data further provides evidence that the blood and nasal mucosa function as separate immune compartments with shared roles in the pathogenesis of AR.

Overall, investigation of the peripheral blood and nasal mucosa with the NanoString nCounter system revealed distinct gene expression profiles in our AR cohort compared with controls. Notably, the peripheral blood DEGs represented a large proportion of the total immune genes, indicating the unique nature of the systemic immune system in AR compared with controls. The shared DEGs in the nasal lysate and blood samples and the associations between clinical markers in blood and gene expression in the nasal mucosa, points to a strong interaction between these immune compartments in the pathogenesis of AR. The AR‐specific genes and pathways identified in this study have the potential to be targeted in new drug therapies for the reduction of AR symptoms and as markers to evaluate the effectiveness of systemic and topical AR drugs.

## AUTHOR CONTRIBUTIONS

*Design*: Annabelle M. Watts, Nicholas P. West, Amanda J. Cox. *Data collection*; Annabelle M. Watts, Amanda J. Cox. *Data analysis*: Annabelle M. Watts, Nicholas P. West. *Review of outcomes*: Allan W. Cripps, Peter K. Smith. *Drafting of manuscript*: Annabelle M. Watts Amanda J. Cox. *Manuscript revision and approval*: all authors.

## Supporting information

Supplementary information.

Supplementary information.

## Data Availability

The data that support the findings of this study are available on request from the corresponding author. The data are not publicly available due to privacy or ethical restrictions.
