# Supplementary material for: Adult allergic rhinitis sufferers have unique nasal mucosal and peripheral blood immune gene expression profiles: A case–control study
Source: Immun Inflamm Dis. 2021 Oct 12;10(1):78–92. doi: 10.1002/iid3.545 (PMC8669689; doi:10.1002/iid3.545)
Supplement: Supplementary file 1 — Supplementary information. [file IID3-10-78-s001.docx]

**Supplementary Tables**

Supplementary Table 1: Clinical and demographic features of the study cohort for participants whose nasal lysate samples met the quality control guidelines and were included in the gene expression analysis.

|  | **All**  **mean ± SD** | **AR**  **mean ± SD** | **CG**  **mean ± SD** | **P-value** |
| --- | --- | --- | --- | --- |
| n | 58 | 37 | 21 | - |
| Age (years) | 37.34 ± 12.12 | 38.19 ± 12.92 | 35.83 ± 10.71 | 0.481 |
| Sex (M/F) | 24/34 (59%) | 14/23 (62%) | 10/11 (52%) | 0.467 |
| Height (cm) | 173.11 ± 9.39 | 171.38 ± 9.38 | 176.15 ± 8.82 | 0.062 |
| Weight (kg) | 76.12 ± 17.06 | 75.54 ± 15.75 | 77.14 ± 19.54 | 0.733 |
| BMI (kg/m^2^) | 25.21 ± 4.30 | 25.54 ± 3.99 | 24.61 ± 4.83 | 0.430 |
| Ethnicity (% Caucasian) | 86% | 81% | 95% | 0.133 |
| **Immune measures** |  |  |  |  |
| White cell count (x10^9^/L) | 6.31 ± 1.52 | 6.65 ± 1.58 | 5.73 ± 1.24 | 0.026 |
| Lymphocytes (x10^9^/L) | 2.05 ± 0.67 | 2.16 ± 0.70 | 1.86 ± 0.58 | 0.093 |
| Eosinophils (x10^9^/L) | 0.31 ± 0.29 | 0.42 ± 0.31 | 0.11 ± 0.09 | <0.00001 |
| Neutrophils (x10^9^/L) | 3.41 ± 1.00 | 3.49 ± 1.02 | 3.26 ± 0.98 | 0.411 |
| Basophils (x10^9^/L) | 0.05 ± 0.04 | 0.06 ± 0.04 | 0.03 ± 0.03 | 0.032 |
| ESR (mm/hr) | 8.69 ± 8.87 | 9.22 ± 9.36 | 7.76 ± 8.08 | 0.533 |

n, number; AR, allergic rhinitis; CG, Control Group; M, Male; F, Female; cm, centimetre; kg, kilogram; m, metre; %, percentage; L, litre; mm, millimetre; hr, hour

Supplementary table 2: Disease characteristics of the of the study cohort for participants whose nasal lysate samples met the quality control guidelines and were included in the gene expression analysis.

| **Disease characteristic** | **AR (mean ± SD)** |
| --- | --- |
| *Allergen sensitivity* |  |
| Co-allergy to dust mites and pollen (%) | 59% |
| Dust mite only (%) | 41% |
| IgE *D. pteronyssinus* (kU/L) | 24.50 ± 33.20 |
| IgE *D. farinae* (kU/L) | 20.62 ± 31.00 |
| IgE grass pollen mix (kU/L) | 8.39 ± 22.24 |
| *IgG4* *D. pteronyssinus* (kU/L) | 0.48 ± 0.49 |
| *IgG4 D. farinae* (kU/L) | 0.39 ± 0.37 |
| *IgG4* grass pollen mix (kU/L) | 0.86 ± 0.68 |
| *Symptom severity* |  |
| Total Nasal Symptom Score (0-12 U) | 5.35 ± 3.16 |
| Total Ocular Symptom Score (0-9 U) | 2.59 ± 2.30 |
| Mini rhinoconjunctivitis quality of life score (0-6 U) | 2.77 ± 1.11 |
| Other Allergic Rhinitis Symptom Score (0-12 U) | 3.99 ± 3.23 |
| Visual Analogue Scale (0-100 mm) | 53.22 ± 27.46 |

AR, allergic rhinitis; %, percentage; kU, kilounit; L, Litre; U, unit; mm, millimetre

Supplementary Table 3. Differentially expressed genes in blood samples padjust <0.05

| **Gene** | **Log2 fold change** | **Linear fold change** | **Lower confidence limit (log2)** | **Upper confidence limit (log2)** | **P-value** | **P adjust** |
| --- | --- | --- | --- | --- | --- | --- |
| MAP2K1 | -0.417 | 0.749 | -0.512 | -0.322 | 1.84 x 10^-12^ | 9.86 x 10^-10^ |
| TBK1 | -0.608 | 0.656 | -0.769 | -0.447 | 2.90 x 10^-10^ | 5.18 x 10^-8^ |
| PTGDR2 | 1.730 | 3.310 | 1.270 | 2.180 | 2.78 x 10^-10^ | 5.18 x 10^-8^ |
| CD83 | 0.979 | 1.970 | 0.681 | 1.280 | 1.53 x 10^-8^ | 1.65 x 10^-6^ |
| CD164 | -0.203 | 0.869 | -0.266 | -0.140 | 2.47 x 10^-8^ | 2.21 x 10^-6^ |
| CD24 | 1.320 | 2.500 | 0.903 | 1.740 | 4.22 x 10^-8^ | 3.24 x 10^-6^ |
| IL2RA | 0.946 | 1.930 | 0.601 | 1.290 | 1.06 x 10^-6^ | 7.12 x 10^-5^ |
| MICA | 0.711 | 1.640 | 0.441 | 0.980 | 2.31 x 10^-6^ | 1.38 x 10^-4^ |
| IL12RB1 | 0.527 | 1.440 | 0.318 | 0.736 | 5.31 x 10^-6^ | 2.85 x 10^-4^ |
| ABCB1 | -0.544 | 0.686 | -0.764 | -0.325 | 7.21 x 10^-6^ | 3.52 x 10^-4^ |
| LILRA1 | -0.484 | 0.715 | -0.681 | -0.287 | 8.71 x 10^-6^ | 3.90 x 10^-4^ |
| HMGB1 | -0.177 | 0.884 | -0.250 | -0.104 | 1.02 x 10^-5^ | 4.23 x 10^-4^ |
| NCR1 | -0.674 | 0.627 | -0.958 | -0.391 | 1.51 x 10^-5^ | 5.79 x 10^-4^ |
| IFNGR1 | -0.350 | 0.785 | -0.500 | -0.200 | 2.20 x 10^-5^ | 7.40 x 10^-4^ |
| IRF3 | 0.527 | 1.440 | 0.300 | 0.753 | 2.20 x 10^-5^ | 7.40 x 10^-4^ |
| TNFAIP3 | -0.275 | 0.827 | -0.393 | -0.156 | 2.42 x 10^-5^ | 7.65 x 10^-4^ |
| HRH4 | 0.896 | 1.860 | 0.507 | 1.280 | 2.64 x 10^-5^ | 7.88 x 10^-4^ |
| IKBKG | -0.303 | 0.811 | -0.436 | -0.171 | 2.94 x 10^-5^ | 8.31 x 10^-4^ |
| APP | -0.536 | 0.690 | -0.774 | -0.298 | 3.68 x 10^-5^ | 9.08 x 10^-4^ |
| PSEN1 | -0.292 | 0.817 | -0.422 | -0.163 | 3.61 x 10^-5^ | 9.08 x 10^-4^ |
| SPN | -0.486 | 0.714 | -0.705 | -0.267 | 4.69 x 10^-5^ | 1.10 x 10^-3^ |
| MAPK1 | -0.400 | 0.758 | -0.581 | -0.219 | 5.07 x 10^-5^ | 1.13 x 10^-3^ |
| IL6R | -0.353 | 0.783 | -0.515 | -0.190 | 6.79 x 10^-5^ | 1.40 x 10^-3^ |
| STAT5B | -0.267 | 0.831 | -0.390 | -0.144 | 6.60 x 10^-5^ | 1.40 x 10^-3^ |
| TNFRSF13C | 0.625 | 1.540 | 0.333 | 0.916 | 8.08 x 10^-5^ | 1.61 x 10^-3^ |
| CD4 | -0.322 | 0.800 | -0.473 | -0.171 | 8.55 x 10^-5^ | 1.64 x 10^-3^ |
| FCER2 | 0.816 | 1.760 | 0.430 | 1.200 | 9.78 x 10^-5^ | 1.81 x 10^-3^ |
| FYN | -0.187 | 0.878 | -0.278 | -0.097 | 1.31 x 10^-4^ | 2.35 x 10^-3^ |
| ECSIT | -0.410 | 0.752 | -0.610 | -0.211 | 1.44 x 10^-4^ | 2.50 x 10^-3^ |
| TNFRSF10B | -0.355 | 0.782 | -0.528 | -0.182 | 1.50 x 10^-4^ | 2.52 x 10^-3^ |
| CTSS | -0.298 | 0.813 | -0.445 | -0.152 | 1.60 x 10^-4^ | 2.61 x 10^-3^ |
| EWSR1 | -0.119 | 0.921 | -0.178 | -0.061 | 1.67 x 10^-4^ | 2.63 x 10^-3^ |
| CD36 | -0.480 | 0.717 | -0.720 | -0.240 | 2.13 x 10^-4^ | 3.02 x 10^-3^ |
| NOTCH1 | -0.440 | 0.737 | -0.660 | -0.221 | 2.04 x 10^-4^ | 3.02 x 10^-3^ |
| MAP2K4 | -0.326 | 0.798 | -0.489 | -0.163 | 2.13 x 10^-4^ | 3.02 x 10^-3^ |
| CAMP | 1.020 | 2.020 | 0.509 | 1.520 | 2.04 x 10^-4^ | 3.02 x 10^-3^ |
| IL5RA | 1.210 | 2.310 | 0.601 | 1.820 | 2.26 x 10^-4^ | 3.11 x 10^-3^ |
| TLR2 | -0.448 | 0.733 | -0.676 | -0.221 | 2.59 x 10^-4^ | 3.23 x 10^-3^ |
| MAPK8 | -0.350 | 0.785 | -0.527 | -0.173 | 2.48 x 10^-4^ | 3.23 x 10^-3^ |
| CYBB | -0.347 | 0.786 | -0.522 | -0.171 | 2.53 x 10^-4^ | 3.23 x 10^-3^ |
| IRAK4 | -0.189 | 0.877 | -0.284 | -0.093 | 2.48 x 10^-4^ | 3.23 x 10^-3^ |
| TICAM1 | 0.327 | 1.250 | 0.159 | 0.496 | 3.04 x 10^-4^ | 3.71 x 10^-3^ |
| LRP1 | -0.634 | 0.644 | -0.961 | -0.306 | 3.22 x 10^-4^ | 3.84 x 10^-3^ |
| LCP1 | -0.286 | 0.820 | -0.434 | -0.137 | 3.51 x 10^-4^ | 4.09 x 10^-3^ |
| CD99 | -0.260 | 0.835 | -0.397 | -0.122 | 4.29 x 10^-4^ | 4.80 x 10^-3^ |
| IL1RL1 | 0.908 | 1.880 | 0.428 | 1.390 | 4.26 x 10^-4^ | 4.80 x 10^-3^ |
| CD79A | 0.524 | 1.440 | 0.245 | 0.803 | 4.61 x 10^-4^ | 5.05 x 10^-3^ |
| NUP107 | 0.198 | 1.150 | 0.092 | 0.304 | 4.94 x 10^-4^ | 5.30 x 10^-3^ |
| ITGA5 | -0.236 | 0.849 | -0.364 | -0.109 | 5.41 x 10^-4^ | 5.69 x 10^-3^ |
| TNFRSF1B | -0.325 | 0.798 | -0.502 | -0.148 | 6.07 x 10^-4^ | 6.27 x 10^-3^ |
| ITGB1 | -0.321 | 0.800 | -0.501 | -0.141 | 8.40 x 10^-4^ | 8.19 x 10^-3^ |
| IKBKE | 0.225 | 1.170 | 0.099 | 0.351 | 8.54 x 10^-4^ | 8.19 x 10^-3^ |
| C3AR1 | 0.601 | 1.520 | 0.264 | 0.938 | 8.37 x 10^-4^ | 8.19 x 10^-3^ |
| IDO1 | 0.871 | 1.830 | 0.384 | 1.360 | 8.16 x 10^-4^ | 8.19 x 10^-3^ |
| CD68 | -0.346 | 0.787 | -0.543 | -0.149 | 1.01 x 10^-3^ | 9.43 x 10^-3^ |
| ITGAE | -0.243 | 0.845 | -0.382 | -0.104 | 1.03 x 10^-3^ | 9.43 x 10^-3^ |
| LTF | 1.170 | 2.250 | 0.501 | 1.830 | 1.04 x 10^-3^ | 9.43 x 10^-3^ |
| SMAD2 | -0.147 | 0.903 | -0.232 | -0.063 | 1.09 x 10^-3^ | 9.63 x 10^-3^ |
| CD84 | 0.237 | 1.180 | 0.101 | 0.373 | 1.08 x 10^-3^ | 9.63 x 10^-3^ |
| F13A1 | -0.671 | 0.628 | -1.060 | -0.284 | 1.14 x 10^-3^ | 9.86 x 10^-3^ |
| RNASE3 | 1.100 | 2.140 | 0.461 | 1.740 | 1.24 x 10^-3^ | 1.05 x 10^-2^ |
| PF4 | -0.659 | 0.633 | -1.040 | -0.274 | 1.32 x 10^-3^ | 1.06 x 10^-2^ |
| TBX21 | -0.442 | 0.736 | -0.701 | -0.184 | 1.33 x 10^-3^ | 1.06 x 10^-2^ |
| IL6ST | -0.278 | 0.825 | -0.440 | -0.115 | 1.33 x 10^-3^ | 1.06 x 10^-2^ |
| FLT3LG | 0.239 | 1.180 | 0.100 | 0.379 | 1.27 x 10^-3^ | 1.06 x 10^-2^ |
| IL1R2 | -0.462 | 0.726 | -0.734 | -0.190 | 1.40 x 10^-3^ | 1.11 x 10^-2^ |
| TNFRSF1A | -0.254 | 0.839 | -0.404 | -0.104 | 1.47 x 10^-3^ | 1.13 x 10^-2^ |
| NFKBIA | -0.232 | 0.852 | -0.369 | -0.095 | 1.50 x 10^-3^ | 1.13 x 10^-2^ |
| INPP5D | -0.152 | 0.900 | -0.242 | -0.062 | 1.49 x 10^-3^ | 1.13 x 10^-2^ |
| LAMP2 | -0.290 | 0.818 | -0.464 | -0.116 | 1.69 x 10^-3^ | 1.24 x 10^-2^ |
| UBC | -0.159 | 0.896 | -0.254 | -0.064 | 1.68 x 10^-3^ | 1.24 x 10^-2^ |
| STAT3 | -0.286 | 0.820 | -0.459 | -0.113 | 1.84 x 10^-3^ | 1.33 x 10^-2^ |
| EP300 | -0.153 | 0.899 | -0.245 | -0.060 | 1.87 x 10^-3^ | 1.34 x 10^-2^ |
| CD247 | -0.222 | 0.857 | -0.357 | -0.087 | 1.94 x 10^-3^ | 1.37 x 10^-2^ |
| MR1 | 0.345 | 1.270 | 0.133 | 0.557 | 2.17 x 10^-3^ | 1.49 x 10^-2^ |
| CCR3 | 0.659 | 1.580 | 0.254 | 1.060 | 2.15 x 10^-3^ | 1.49 x 10^-2^ |
| ITGB2 | -0.208 | 0.866 | -0.336 | -0.080 | 2.20 x 10^-3^ | 1.50 x 10^-2^ |
| LAMP1 | -0.196 | 0.873 | -0.317 | -0.075 | 2.29 x 10^-3^ | 1.54 x 10^-2^ |
| PECAM1 | -0.265 | 0.832 | -0.430 | -0.101 | 2.37 x 10^-3^ | 1.57 x 10^-2^ |
| ITGAM | -0.257 | 0.837 | -0.417 | -0.097 | 2.42 x 10^-3^ | 1.59 x 10^-2^ |
| LILRB2 | -0.304 | 0.810 | -0.495 | -0.113 | 2.66 x 10^-3^ | 1.72 x 10^-2^ |
| JUN | 0.526 | 1.440 | 0.195 | 0.858 | 2.74 x 10^-3^ | 1.75 x 10^-2^ |
| CCL5 | -0.334 | 0.793 | -0.547 | -0.121 | 3.09 x 10^-3^ | 1.87 x 10^-2^ |
| CTSH | -0.306 | 0.809 | -0.501 | -0.110 | 3.11 x 10^-3^ | 1.87 x 10^-2^ |
| LILRB1 | -0.292 | 0.817 | -0.478 | -0.106 | 3.08 x 10^-3^ | 1.87 x 10^-2^ |
| CCR2 | -0.264 | 0.833 | -0.431 | -0.096 | 2.95 x 10^-3^ | 1.87 x 10^-2^ |
| HLA-E | -0.155 | 0.898 | -0.254 | -0.056 | 3.01 x 10^-3^ | 1.87 x 10^-2^ |
| RIPK2 | 0.241 | 1.180 | 0.086 | 0.396 | 3.30 x 10^-3^ | 1.97 x 10^-2^ |
| LILRB3 | -0.334 | 0.793 | -0.551 | -0.117 | 3.57 x 10^-3^ | 2.10 x 10^-2^ |
| TFRC | -0.452 | 0.731 | -0.751 | -0.152 | 4.27 x 10^-3^ | 2.50 x 10^-2^ |
| IL13RA1 | -0.298 | 0.813 | -0.498 | -0.099 | 4.53 x 10^-3^ | 2.59 x 10^-2^ |
| GPI | 0.195 | 1.140 | 0.065 | 0.325 | 4.52 x 10^-3^ | 2.59 x 10^-2^ |
| IRF5 | 0.391 | 1.310 | 0.129 | 0.652 | 4.71 x 10^-3^ | 2.66 x 10^-2^ |
| LTBR | -0.338 | 0.791 | -0.565 | -0.110 | 4.85 x 10^-3^ | 2.71 x 10^-2^ |
| CD3G | -0.223 | 0.857 | -0.379 | -0.068 | 6.48 x 10^-3^ | 3.59 x 10^-2^ |
| TNFRSF8 | 0.358 | 1.280 | 0.106 | 0.611 | 6.97 x 10^-3^ | 3.82 x 10^-2^ |
| ATG5 | -0.121 | 0.920 | -0.206 | -0.036 | 7.12 x 10^-3^ | 3.86 x 10^-2^ |
| SOCS1 | 0.441 | 1.360 | 0.128 | 0.754 | 7.36 x 10^-3^ | 3.95 x 10^-2^ |
| FUT7 | -0.322 | 0.800 | -0.551 | -0.093 | 7.53 x 10^-3^ | 4.00 x 10^-2^ |
| YTHDF2 | -0.129 | 0.915 | -0.221 | -0.037 | 7.69 x 10^-3^ | 4.04 x 10^-2^ |
| OAS3 | 0.883 | 1.840 | 0.253 | 1.510 | 7.76 x 10^-3^ | 4.04 x 10^-2^ |
| TNFSF4 | -0.467 | 0.723 | -0.806 | -0.128 | 8.71 x 10^-3^ | 4.49 x 10^-2^ |
| CSF3R | -0.249 | 0.842 | -0.429 | -0.068 | 8.79 x 10^-3^ | 4.49 x 10^-2^ |
| SYK | -0.227 | 0.854 | -0.392 | -0.062 | 8.92 x 10^-3^ | 4.52 x 10^-2^ |
| ADORA2A | -0.326 | 0.798 | -0.563 | -0.088 | 9.11 x 10^-3^ | 4.54 x 10^-2^ |
| IFNAR1 | -0.272 | 0.828 | -0.470 | -0.073 | 9.17 x 10^-3^ | 4.54 x 10^-2^ |
| CHUK | -0.193 | 0.875 | -0.335 | -0.052 | 9.38 x 10^-3^ | 4.54 x 10^-2^ |
| RUNX3 | 0.247 | 1.190 | 0.066 | 0.428 | 9.38 x 10^-3^ | 4.54 x 10^-2^ |
| ISG15 | 0.785 | 1.720 | 0.211 | 1.360 | 9.23 x 10^-3^ | 4.54 x 10^-2^ |
| CD79B | 0.312 | 1.240 | 0.083 | 0.541 | 9.62 x 10^-3^ | 4.61 x 10^-2^ |
| ITGAL | -0.156 | 0.898 | -0.270 | -0.041 | 9.75 x 10^-3^ | 4.63 x 10^-2^ |
| CX3CR1 | -0.326 | 0.798 | -0.567 | -0.085 | 1.00 x 10^-2^ | 4.72 x 10^-2^ |
| PPBP | -0.543 | 0.686 | -0.946 | -0.141 | 1.02 x 10^-2^ | 4.76 x 10^-2^ |

Supplementary Table 4: Differentially expressed genes in nasal lysate samples padjust <0.05

| **Gene** | **Log2 fold change** | **Linear fold change** | **Lower confidence limit (log2)** | **Upper confidence limit (log2)** | **P-value** | **P adjust** |
| --- | --- | --- | --- | --- | --- | --- |
| CCL17 | 2.840 | 7.140 | 2.030 | 3.640 | 4.41 x 10^-9^ | 2.51 x 10^-6^ |
| CCL26 | 2.720 | 6.610 | 1.820 | 3.630 | 2.39 x 10^-7^ | 5.13 x 10^-5^ |
| TPSAB1 | 3.080 | 8.450 | 2.050 | 4.110 | 2.71 x 10^-7^ | 5.13 x 10^-5^ |
| PTGS1 | 2.410 | 5.300 | 1.490 | 3.330 | 4.01 x 10^-6^ | 4.57 x 10^-4^ |
| IL1RL1 | 3.410 | 10.700 | 1.970 | 4.860 | 2.14 x 10^-5^ | 2.02 x 10^-3^ |
| CD1A | 2.460 | 5.520 | 1.400 | 3.530 | 3.05 x 10^-5^ | 2.48 x 10^-3^ |
| CCND3 | 1.130 | 2.190 | 0.570 | 1.690 | 2.36 x 10^-4^ | 1.68 x 10^-2^ |
| PPBP | -1.650 | 0.320 | -2.510 | -0.780 | 4.60 x 10^-4^ | 2.91 x 10^-2^ |
| IL18R1 | 1.960 | 3.890 | 0.880 | 3.040 | 7.53 x 10^-4^ | 3.89 x 10^-2^ |
| CD1C | 1.560 | 2.960 | 0.680 | 2.440 | 9.70 x 10^-4^ | 4.04 x 10^-2^ |
| CTSH | -0.570 | 0.680 | -0.890 | -0.250 | 1.01 x 10^-3^ | 4.04 x 10^-2^ |
| FLT3LG | 1.160 | 2.230 | 0.500 | 1.810 | 1.06 x 10^-3^ | 4.04 x 10^-2^ |
| RUNX3 | 1.680 | 3.210 | 0.740 | 2.630 | 9.63 x 10^-4^ | 4.04 x 10^-2^ |
| PTGDR2 | 2.440 | 5.430 | 1.040 | 3.840 | 1.17 x 10^-3^ | 4.15 x 10^-2^ |
| IL33 | -1.370 | 0.390 | -2.220 | -0.520 | 2.61 x 10^-3^ | 7.14 x 10^-2^ |
| JUN | -0.830 | 0.560 | -1.340 | -0.310 | 2.63 x 10^-3^ | 7.14 x 10^-2^ |
| TXNIP | 0.890 | 1.850 | 0.340 | 1.440 | 2.45 x 10^-3^ | 7.14 x 10^-2^ |
| KLRB1 | -1.470 | 0.360 | -2.390 | -0.550 | 2.78 x 10^-3^ | 7.20 x 10^-2^ |
| CXCR3 | 1.380 | 2.600 | 0.510 | 2.250 | 3.06 x 10^-3^ | 7.58 x 10^-2^ |
| CARD9 | 1.290 | 2.450 | 0.460 | 2.120 | 3.50 x 10^-3^ | 8.31 x 10^-2^ |
| CYSLTR1 | 1.600 | 3.030 | 0.550 | 2.650 | 4.29 x 10^-3^ | 9.39 x 10^-2^ |
| SOCS1 | 1.640 | 3.110 | 0.550 | 2.730 | 4.71 x 10^-3^ | 9.92 x 10^-2^ |
| ATG16L1 | 0.390 | 1.310 | 0.130 | 0.650 | 5.09 x 10^-3^ | 1.03 x 10^-1^ |
| IFI27 | -0.880 | 0.540 | -1.490 | -0.280 | 5.72 x 10^-3^ | 1.07 x 10^-1^ |
| TNFSF13 | 0.720 | 1.640 | 0.230 | 1.200 | 5.83 x 10^-3^ | 1.07 x 10^-1^ |
| PF4 | 1.340 | 2.520 | 0.410 | 2.260 | 6.54 x 10^-3^ | 1.16 x 10^-1^ |
| IRAK4 | 0.650 | 1.570 | 0.190 | 1.100 | 7.71 x 10^-3^ | 1.33 x 10^-1^ |
| RRAD | -0.950 | 0.520 | -1.620 | -0.270 | 8.01 x 10^-3^ | 1.34 x 10^-1^ |
| NFATC2 | 1.070 | 2.100 | 0.300 | 1.840 | 8.38 x 10^-3^ | 1.36 x 10^-1^ |
| C3 | -0.990 | 0.500 | -1.720 | -0.270 | 9.46 x 10^-3^ | 1.50 x 10^-1^ |
| CCR7 | 1.850 | 3.600 | 0.480 | 3.220 | 1.07 x 10^-2^ | 1.57 x 10^-1^ |
| MRC1 | 1.810 | 3.500 | 0.430 | 3.190 | 1.30 x 10^-2^ | 1.76 x 10^-1^ |
| TFEB | 0.840 | 1.790 | 0.190 | 1.480 | 1.36 x 10^-2^ | 1.80 x 10^-1^ |
| PIK3CG | 1.350 | 2.550 | 0.310 | 2.390 | 1.40 x 10^-2^ | 1.81 x 10^-1^ |
| SMPD3 | 1.200 | 2.300 | 0.270 | 2.130 | 1.43 x 10^-2^ | 1.81 x 10^-1^ |
| IKBKE | -0.450 | 0.730 | -0.800 | -0.100 | 1.47 x 10^-2^ | 1.82 x 10^-1^ |
| TNFSF12 | 1.100 | 2.140 | 0.240 | 1.950 | 1.51 x 10^-2^ | 1.82 x 10^-1^ |
| PAFAH2 | 0.680 | 1.610 | 0.140 | 1.230 | 1.64 x 10^-2^ | 1.94 x 10^-1^ |
| IL16 | 1.300 | 2.460 | 0.260 | 2.340 | 1.79 x 10^-2^ | 2.07 x 10^-1^ |
| RNASE3 | 1.400 | 2.630 | 0.270 | 2.520 | 1.82 x 10^-2^ | 2.07 x 10^-1^ |
| IL3RA | 2.230 | 4.690 | 0.410 | 4.050 | 1.96 x 10^-2^ | 2.10 x 10^-1^ |
| TYK2 | 0.650 | 1.570 | 0.120 | 1.180 | 2.02 x 10^-2^ | 2.13 x 10^-1^ |
| CD36 | 0.680 | 1.600 | 0.120 | 1.240 | 2.16 x 10^-2^ | 2.20 x 10^-1^ |
| CLEC4C | 1.760 | 3.390 | 0.290 | 3.240 | 2.29 x 10^-2^ | 2.25 x 10^-1^ |
| LRP1 | -0.540 | 0.690 | -0.990 | -0.080 | 2.42 x 10^-2^ | 2.30 x 10^-1^ |
| SCGB1A1 | -0.850 | 0.560 | -1.570 | -0.120 | 2.54 x 10^-2^ | 2.36 x 10^-1^ |
| SMAD3 | -0.350 | 0.790 | -0.650 | -0.050 | 2.57 x 10^-2^ | 2.36 x 10^-1^ |
| CD40 | -0.720 | 0.610 | -1.340 | -0.100 | 2.68 x 10^-2^ | 2.42 x 10^-1^ |
| CCR3 | 2.170 | 4.510 | 0.250 | 4.100 | 3.11 x 10^-2^ | 2.59 x 10^-1^ |
| CREBBP | 0.640 | 1.550 | 0.080 | 1.200 | 3.03 x 10^-2^ | 2.59 x 10^-1^ |
| CX3CL1 | -1.010 | 0.500 | -1.900 | -0.120 | 3.10 x 10^-2^ | 2.59 x 10^-1^ |
| NCAM1 | 1.000 | 2.000 | 0.120 | 1.880 | 3.01 x 10^-2^ | 2.59 x 10^-1^ |
| REPS1 | 0.270 | 1.210 | 0.030 | 0.520 | 3.14 x 10^-2^ | 2.59 x 10^-1^ |
| SERPINB2 | 0.640 | 1.550 | 0.070 | 1.200 | 3.11 x 10^-2^ | 2.59 x 10^-1^ |
| TRAF3 | -0.840 | 0.560 | -1.590 | -0.090 | 3.24 x 10^-2^ | 2.63 x 10^-1^ |
| MAP2K2 | 0.310 | 1.240 | 0.030 | 0.590 | 3.62 x 10^-2^ | 2.90 x 10^-1^ |
| CYLD | 0.670 | 1.590 | 0.030 | 1.300 | 4.34 x 10^-2^ | 3.21 x 10^-1^ |
| EGR1 | 1.040 | 2.050 | 0.060 | 2.010 | 4.19 x 10^-2^ | 3.21 x 10^-1^ |
| GZMB | 1.420 | 2.680 | 0.080 | 2.760 | 4.30 x 10^-2^ | 3.21 x 10^-1^ |
| MAPK1 | 0.560 | 1.470 | 0.040 | 1.080 | 4.10 x 10^-2^ | 3.21 x 10^-1^ |
| TRAF6 | 0.370 | 1.290 | 0.020 | 0.720 | 4.27 x 10^-2^ | 3.21 x 10^-1^ |
| CD68 | -0.720 | 0.610 | -1.400 | -0.030 | 4.42 x 10^-2^ | 3.23 x 10^-1^ |
| VEGFA | -0.870 | 0.550 | -1.690 | -0.040 | 4.53 x 10^-2^ | 3.24 x 10^-1^ |
