# Supplementary material for: Adult allergic rhinitis sufferers have unique nasal mucosal and peripheral blood immune gene expression profiles: A case–control study
Source: Immun Inflamm Dis. 2021 Oct 12;10(1):78–92. doi: 10.1002/iid3.545 (PMC8669689; doi:10.1002/iid3.545)
Supplement: Supplementary file 2 — Supplementary information. [file IID3-10-78-s002.pdf]

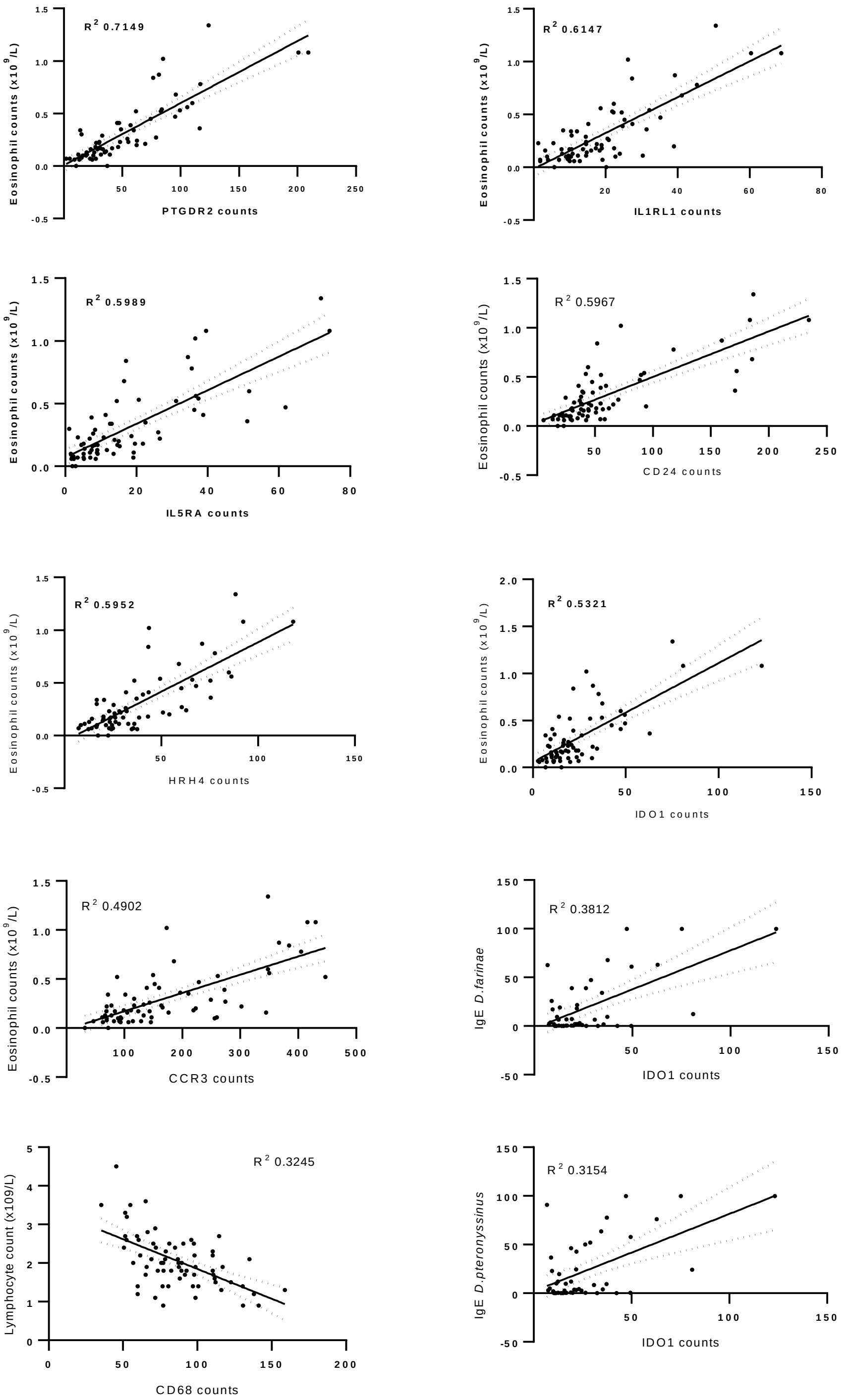

Supplementary Figure 1: Top 10 Individual correlation plots for the differentially expressed genes in blood and clinical factors. Each point represents a sample (filled circles). Clinical factors are on the y axis and gene counts are on the x axis. Lines of best fit are plotted as a solid line with the 95% confidence bands as a dotted line for each correlation. Each correlation was significant ($p<0.05$) and the direction indicates a negative or positive correlation. Correlations for IgE *D.farinae* and IgE *D. pteronyssinus* were conducted on AR samples only (n=45). The remaining correlations were performed with the inclusion of the control group samples (n=69 total).

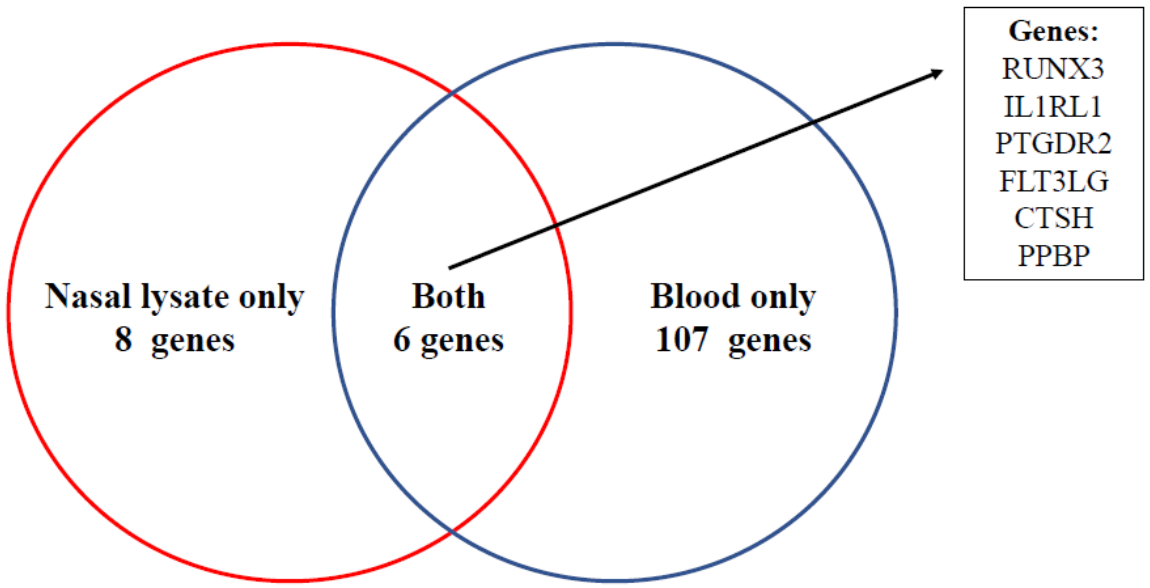

Supplementary Figure 2. Venn diagram displaying the number of DEGs unique to nasal lysate and blood samples and DEGs that were shared between both sample types.
